# Anal Squamous Cell Carcinoma in a Patient with Myasthenia Gravis: Is Immunosuppression the Main Underlying Etiology?

**DOI:** 10.7759/cureus.1845

**Published:** 2017-11-15

**Authors:** Muhammad Masab, Muhammad W Saif

**Affiliations:** 1 Internal Medicine, Albert Einstein medical center; 2 Hematology/Oncology, Tufts Medical Center

**Keywords:** anal cancer, immunosuppression, myasthenia gravis, thymoma, transplantation, anal hpv, human papilloma virus (hpv)

## Abstract

Patients who are immunocompromised by diseases such as human immunodeficiency virus (HIV) infection are more prone to develop some malignancies such as Kaposi's sarcoma and central nervous system (CNS) lymphomas. Historically, anal squamous cell carcinoma (SCC) was also included on the list as an acquired immunodeficiency syndrome (AIDs)-defining cancer.  Similarly, compromised immune disorders including severe immunosuppression, haematologic malignancies, and solid organ transplantation have been identified as important risk factors for the development of anal SCC. Review of the medical literature showed only sporadic cases of anal SCC in patients with pre-existing myasthenia gravis (MG), with or without thymoma.  We present here a case of anal SCC in a patient with several years history of MG who was receiving intravenous immunoglobulin (IVIG).  We believe this association is explained by the autoimmune nature of the disease and the use of immunosuppressive medications to treat it. To further support our case, we also present a review of the literature associating anal SCC with MG.

## Introduction

Anal squamous cell carcinoma (SCC) is an uncommon malignancy and accounts for only a small percentage (4%) of all cancers of the lower gastrointestinal (GI) tract [1]. The risk of development of anal SCC is related to persons engaging in receptive anal intercourse or persons with a high lifetime number of sexual partners and human papillomavirus (HPV) infection. HPV infection is considered a necessary step in the carcinogenesis of anal SCC [2]. Additionally, other compromised immune disorders such as hematologic malignancies, solid organ transplantation, and human immunodeficiency virus (HIV) infection are also associated with increased susceptibility to the development of anal SCC [3-4]. Anecdotally, patients with myasthenia gravis (MG) with or without thymoma have been reported in patients with risk of developing extrathymic tumors, such as colon cancer, breast cancer, lymphoma and very rarely anal SCC [5-6]. We present here a unique case related to the rare association between MG and anal SCC and review the literature. We consider that this association is clinically relevant because anal cancer is usually a curable tumor and recognition of this malignancy is, therefore, very important.  

## Case presentation

A 54-year-old woman with past medical history of MG for several years presented to us with the new diagnosis of anal SCC. Fifteen years ago, she presented with double vision, dysphagia, and proximal weakness of extremities. Electromyography (EMG) and positive anti-acetylcholine receptor (AchR) antibody confirmed the diagnosis of MG.  She was started on mycophenolate, pyridostigmine, and prednisone with some improvement of her symptoms. A follow-up computerized tomography (CT) scan of the chest revealed a six-centimeter anterior mediastinal mass that was surgically resected and pathology revealed thymoma. She received six weeks of postoperative radiation therapy to her chest. She continued to be monitored regularly by her neurologist and receive intravenous immunoglobulin (IVIG) periodically for exacerbation of her symptoms or myasthenia crisis.

She was referred to us due to decreased appetite, diarrhea, and 10-pound weight loss over a period of eight weeks. Given her history of thymoma and MG, our clinical suspicion for a malignancy was high. CT scan of the abdomen and magnetic resonance imaging (MRI) of the pelvis showed an infiltrative mass in the anus. Colonoscopy was performed with the biopsy of the anal canal that revealed moderately differentiated invasive SCC. Immunohistostaining was positive for HPV. She was diagnosed with stage IIIB anal SCC based on endoscopic ultrasound (EUS) and radiological imaging with an MRI and CT scans.

After a multidisciplinary evaluation by a medical oncologist, radiation oncologist, and a surgeon, she was treated with concurrent chemo-radiotherapy consisting of fluorouracil (5-FU) and mitomycin-C based on Radiation Therapy Oncology Group (RTOG) study no. 8314 [7]. Surveillance examination, MRI and positron emission tomography (PET) scan performed three months and six months post-treatment showed complete resolution of the soft tissue mass with no evidence of tumor. 

## Discussion

In patients with autoimmune diseases like MG, immune function can be compromised because of intrinsic alteration in the immune system, immunosuppressive therapies, or both. Consequently, patients with autoimmune disease may be at increased risk of anal SCC [5-6]. We believe that our present case falls into this criteria.

MG is an autoimmune disease characterized by fluctuating degree of weakness in ocular, bulbar, respiratory, and limb muscles. It is caused by auto-antibody mediated, T cell-dependent immunological attack on AchR on the post-synaptic neuronal membrane of the neuromuscular junction. MG patients are at an increased risk of developing cancers because of their weak immune system. Their immune function is compromised by the autoimmune nature of the disease as well as the use of immunosuppressive medications in its treatment. Moreover, it is well known that MG patients (30-50% of cases) frequently have benign and malignant thymomas that also increase the risk of extrathymic malignancy [4-6]. 

A close association exists between infection by oncogenic HPV strains and anal SCC  [2]. Epidemiologic studies have shown that up to 93% of anal SCCs are associated with HPV infection. However, the majority of anal HPV infections resolve in a relatively short time indicating that other factors are also involved in the progression from anal HPV infection to cancer. In addition to immune-compromised conditions, including HIV infection, solid organ transplantation, hematologic malignancies, and autoimmune diseases, chronic glucocorticoid therapy for the treatment of autoimmune disease can also predispose to HPV-related anal SCC [3-4].

Data on the association between anal SCC and MG is scarce. Dysregulation of the immune function in autoimmune diseases like MG can potentially lead to cancer development and can help explain the linking of autoimmune mechanisms with cancer. A cohort study done in Denmark showed an increased risk of anal SCC in autoimmune diseases including MG [5]. Hemminki, et al. studied the occurrence of histology-specific GI cancers in patients diagnosed with 33 different autoimmune diseases. They reported that MG was associated with five different GI cancers (upper digestive tract, esophageal SCC, stomach cancer, and colorectal cancer) with standardized incident ratios ranging from 1.35 to 2.78 [6].

There are many case reports and retrospective studies illustrating the association of secondary neoplasms in MG patients with thymoma [8-10]. The pathogenetic link between thymoma and carcinoma has not yet been completely clarified. It is suggested that development of thymoma, implies a defect in thymic epithelium that hinders T-cell development, leading to immune defects and a higher incidence of cancer. The prevalence rates are as high as 31%. The most frequent secondary primary neoplasms are colorectal cancer and thyroid cancer. Weksler, et al. performed a retrospective analysis of 2,171 patients with thymoma and reported that 306 (14.1%) of patients had extrathymic primary cancers, most commonly lymphoma, breast cancer, prostate cancer, lung cancer, and colorectal cancer [8]. Extrathymic neoplasms were diagnosed before the diagnosis of thymoma in 88 patients and after the diagnosis of thymoma in 206 patients. They concluded that incidence of extrathymic neoplasms is significantly higher in patients with thymoma than in the general population and occurs both before and after the diagnosis of thymoma irrespective of the radiation therapy or surgical treatment provided for thymoma [8]. Masaoka, et al. reported a series of 392 patients who underwent thymectomy for MG; 102 patients had thymomas. Nine patients experienced malignancies; eight of the nine (7.8%) had a diagnosis of thymoma [9]. Some other retrospective studies and cancer registry surveys showed similar findings indicating that extrathymic malignancy can be diagnosed up to 10 years after the diagnosis of thymoma and that the most common tumors were GI and respiratory cancers [10].

Interestingly, our patient fulfills all of the criteria as she has a history of MG over 15 years and was diagnosed with thymoma as well. In addition, she initially received immunosuppressive medications, including steroids, but IVIG only occasionally. In addition, our patient developed anal SCC, a GI tumor, considered to be the most common site in these patients per the literature review.

## Conclusions

This case is presented to highlight the possible association between MG and anal SCC under the common background of immune dysregulation and presence of thymoma. Further studies are needed to clarify the role of the immune system in cancer development associated with MG. The physicians should always consider the possibility of a malignancy in patients with MG. The occurrence of anal SCC with MG and thymoma may be a rare entity, but since anal cancer is usually curable cancer, its recognition and prompt treatment is critical. If similar reports continue to be published in the medical literature, then close, lifetime follow-up of all patients with MG (to allow early detection of this curable cancer) may be considered.
